# What do we know about physical activity interventions in vocational education and training? A systematic review

**DOI:** 10.1186/s12889-020-09093-7

**Published:** 2020-06-22

**Authors:** Eva Grüne, Johanna Popp, Johannes Carl, Klaus Pfeifer

**Affiliations:** Department of Sport Science and Sport, Friedrich-Alexander University Erlangen-Nürnberg, Gebbertstraße 123b, 91058 Erlangen, Germany

**Keywords:** Physical activity, Health promotion, Adolescence, Apprenticeship, Students, School, Workplace

## Abstract

**Background:**

Although the health benefits of physical activity (PA) are well known, young people’s level of PA is often insufficient and tends to decline in adolescence. Numerous studies have investigated the effectiveness of PA-promoting interventions among young people, but none have reviewed the effectiveness of PA interventions in the vocational education and training (VET) setting. This systematic review aims to (1) synthesize and review the available literature on PA-promoting interventions in VET and (2) examine the effects of these interventions on PA-related outcomes such as PA level, physical fitness, physiological parameters, or psychological factors.

**Methods:**

Five electronic databases were searched for studies involving adolescents aged 15 to 20 years that took place in VET settings and evaluated the effects of interventions with a PA component on PA-related outcomes such as PA level, physical fitness, physiological parameters, or psychological factors. The screening process and the quality assessment were conducted by two independent reviewers; data extraction was conducted by one reviewer and verified by another.

**Results:**

The literature search identified 18,959 articles and 11,282 unique records. After the screening process, nine studies, all coming from European or Asian countries, met the pre-defined eligibility criteria and were included in qualitative analyses. All but two studies reported significant improvements for at least one PA-related outcome. The interventions substantially differed in their development approaches (top-down vs. bottom-up approaches), complexity (multi- vs. single-component), and addressed behavior (multi-behavioral vs. single-behavioral). The most conspicuous finding was that bottom-up approaches tend to improve outcomes at the psychological level and top-down approaches at the physical level. Regarding the interventions’ complexity and addressed behavior, we did not reveal any conclusive results.

**Conclusion:**

This systematic review highlights the varying effects of PA-promoting interventions in VET. Nevertheless, heterogeneous effects, overall weak study quality and availability of studies only from two continents limited our ability to draw clear conclusions about the potentially most effective intervention strategies. Therefore, future research should focus on high-quality studies with long-term follow-ups to make recommendations for practical use.

**Trial registration:**

PROSPERO CRD42018109845.

## Background

The world is facing a high prevalence of physical inactivity among young people. Many adolescents do not meet the recommended guidelines of 60 min of moderate to vigorous physical activity per day (PA) [1–3]. A German study, for example, reports that only 7.5% of girls and 16.0% of boys between the ages of 14 and 17 meet these recommendations [4]. These alarming insights are reinforced by evidence that PA continues to decline between adolescence and young adulthood [5]. On the other hand, PA behavior adopted during childhood and adolescence is likely to continue into adulthood [6]. Incontrovertible evidence indicates lifelong health benefits from a physically active lifestyle [7, 8], underlining the need for actions that promote PA. Furthermore, the period ranging from adolescence to young adulthood is an important one for prevention measures, as many health-related behaviors, such as PA patterns, are learned and consolidated at this stage in life [9, 10]. At the same time, this transitional phase also is marked by some major life challenges. Difficulties in the transition from childhood to adulthood or from school to working life – and, thus, to independence and autonomy – influence health and well-being, putting people at greater risk at this life stage [11].

For a large number of young people who do not pursue higher education after secondary school, vocational education and training (VET) is the first step toward working life. In VET, students acquire the knowledge, skills, and competencies specific to particular occupations to gain optimal professional qualifications [12]. A great deal of heterogeneity exists between national VET systems. In Germany, VET is organized in a dual apprenticeship system combining school-based learning and company-based training. Other countries with well-established apprenticeship systems include Austria, Denmark, and Switzerland. In addition to the dual apprenticeship system, VET globally can be categorized into two other systems: school-based VET following a formal curriculum that combines general and occupation-specific knowledge (e.g., France, Sweden, United States) and informal-based VET outside of formal or general schooling (e.g., India, many African countries) [13, 14]. Accordingly, not only do the various institutions in which VET takes place differ, but so also do the respective curricula and duration of VET programs. Nevertheless, they all are linked by a common goal: preparing young people with skills to enter the labor market [15]. Higher education can also prepare people for the world of work, the difference being that VET is characterized by earlier specialization in a particular occupational field and lower socioeconomic status (SES) [14].

Since low SES in adolescence is a predictor of physical inactivity in adulthood [16] and adolescents attending VET often belong to families with low SES [17], these individuals form a group that is vulnerable to engaging in insufficient PA. As PA has not only health-promoting potential but also positive influence on a person’s work ability [18], PA promotion in VET programs are once again coming to the fore in research and practice [19, 20]. In the light of demographic change and the shortage of skilled workers, it is highly important and societally relevant that the future workforce has a good work ability [21].

While numerous studies have confirmed the effectiveness of PA promotion measures in school, university, and workplace settings [22–26], research is lacking about their effectiveness in the VET context. Notwithstanding, VET is a promising setting for PA promotion, as VET programs have a wide reach among adolescents and young adults, providing the opportunity to raise awareness of PA and health at an early stage of life [20, 27]. Against this backdrop, this systematic review aims to (1) synthesize and review the available literature on PA-promoting interventions in VET and (2) examine interventions’ effects on PA-related outcomes such as PA level, physical fitness, physiological parameters, or psychological factors.

## Methods

This systematic review followed the Preferred Reporting Items for Systematic Reviews and Meta-Analyses (PRISMA) guidelines [28] (see Additional file 1) and was registered prospectively in PROSPERO (CRD42018109845).

### Search strategy

To identify interventions that promote PA in VET, we conducted a literature search to retrieve relevant articles published in English or German languages between January 2000 and August 2018. Due to the recent developments in the field of health promotion and especially workplace health promotion (e.g., Jakarta Declaration on Leading Health Promotion into the twenty-first Century [29], Luxembourg Declaration on Workplace Health Promotion [30]), we limited our search to articles from the year 2000 onwards. The following five electronic databases were searched: PsychINFO, PubMed, Scopus, SPORTDiscus, and Web of Science. The literature search included a combination of keywords related to the setting (e.g., VET), health behavior of interest (i.e., PA), and type of study (e.g., intervention) (see Additional file 2). In addition, we used hand and snowball search methods to ensure that all relevant publications were identified.

### Eligibility criteria

In brief, we included studies on apprentices or students (aged 15 to 20 years, inclusive) in VET settings and evaluated the effects from interventions comprising a PA-promoting component on at least one PA-related outcome such as PA level, physical fitness, physiological parameters, or psychological factors. Due to the different international education systems, we also included studies within comparable settings such as community or junior colleges, as these populations and their educational qualifications are very similar to those of VET. We excluded studies that took place at universities, due to entry requirements, higher educational attainment of students, and academic degrees at universities. The full inclusion and exclusion criteria are outlined in Table 1.

**Table 1:** Eligibility criteria

	Inclusion criteria	Exclusion criteria
**Population**	apprentices or VET students aged between 15 to 20 years (inclusive)	apprentices or VET students younger than 15 or older than 20 years
**Setting**	VET or junior/community college	university, elementary school, primary school, high school, middle school
**Intervention**	single or multi-behavioral interventions aimed at promoting PA (i.e., ≥ 25% PA)	
**Outcome**	PA-related outcomes (e.g., PA level, physical fitness, physiological parameters, psychological factors)	
**Study design**	any kind of intervention study	cross-sectional study, review, validation study
**Publication type**	journal article	
**Publication year**	published between 2000 and 2018	
**Language**	English or German	all other languages

*PA* physical activity, *VET* vocational education and training

### Study selection

First, two independent reviewers (EG, JC) screened all titles and abstracts for eligibility using the pre-specified inclusion and exclusion criteria. In a second step, two authors (EG, JP) independently reviewed the full text of papers that potentially were suitable. If it was not clearly evident on the basis of the published article whether the studies met the eligibility criteria, additional information was requested from the investigators. All discrepancies during the study selection process were resolved through discussions among the research team. Inter-rater reliability in selecting studies for inclusion was measured with Cohen’s kappa coefficient [31].

### Data extraction

To summarize eligible studies’ key points, we used a predefined data extraction form that included details on study characteristics, including author, publication year, study design, target group, setting, participant characteristics (sample size, sex, mean age), intervention characteristics (content, focus, strategy, period), and results. If additional information or clarification of data was required, we contacted the authors and included this data in the data extraction process. Additionally, effect sizes were taken either directly from the paper or computed using an online calculator [32]. Following Cohen’s guidelines [31], the effect size of each study was classified as either trivial (|*d* < 0.2|), small (|d = 0.2|), medium (|*d* = 0.5|), or large (|*d* = 0.8|). One reviewer (EG) conducted the extraction, then a second reviewer (JP) verified these results; discrepancies were resolved through discussions.

### Study quality

All studies that met the inclusion criteria underwent quality assessment using the Effective Public Health Practice Project’s (EPHPP) quality assessment tool for quantitative studies [33, 34], recommended by the Cochrane Handbook for Systematic Reviews of Interventions [35]. Two reviewers (EG, JP) independently assessed the quality of the included studies, with the following domains considered: selection bias, study design, confounders, blinding, data collection methods, withdrawals and dropouts, intervention integrity, and statistical analysis. The first six domains were included in the assessment and rated as strong, moderate, or weak, according to the EPHPP dictionary. In case of discrepancies between raters, consensus was reached through discussions.

## Results

**Fig. 1:**
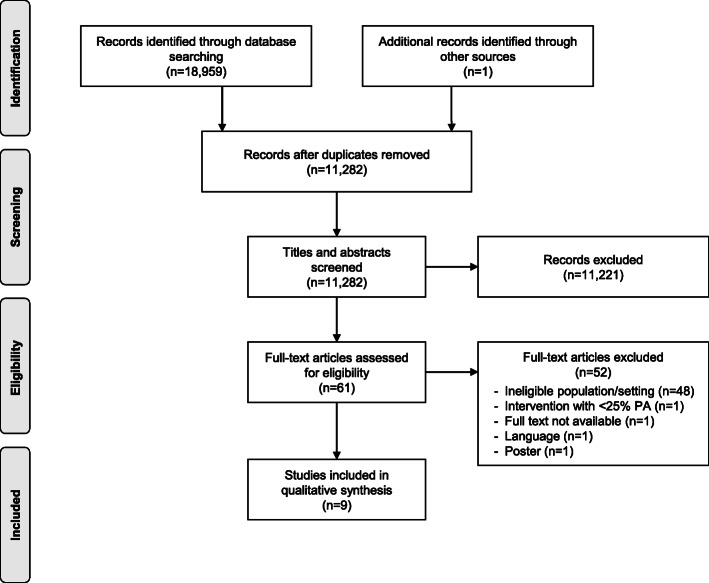
PRISMA flowchart

The PRISMA flowchart in Fig. 1 outlines the search and screening process. The systematic search resulted in 18,959 potentially relevant articles. In addition, we found one article through hand searching. After removing duplicates, 11,282 articles were screened by title and abstract, and 61 full-text articles were assessed for eligibility. The main reasons for exclusion of full-text articles were ineligible study population, i.e., the participants were younger than 15 or older than 20 years, or inappropriate setting, i.e., middle school, university, etc. In total, nine articles met the aforementioned inclusion criteria and were included in the qualitative synthesis [36–44]. Agreement among reviewers was moderate after title and abstract screening (*k* = 0.53), and very good after full-text screening (*k* = 0.87) [45].

### Study quality

Quality ratings are shown in Table 2. With six out of nine studies, the global rating of the majority of studies was weak [36–38, 41–43]. Only one study was rated as strong [40], and two studies were rated as moderate [39, 44]. For the individual EPHPP domains across all studies, blinding was the most weakly rated domain (*n* = 7) [36–38, 41–44]. However, study design and selection bias had no (*n* = 0) and very few (*n* = 2) weak ratings, respectively [36, 37]. Four studies were rated as having strong study design, including randomized controlled trial [44] or cluster randomized controlled trial study designs [39, 40, 42]. The other five studies were rated as moderate with respect to study design strength, with quasi-experimental designs (two groups pre and post [36, 37, 43] or one group pre and post [38, 41]). The other domains differed more in their ratings. While confounders were rated as either strong or weak, data collection method, as well as withdrawals and dropouts, varied similarly in their ratings between weak, moderate, and strong.

**Table 2:** Assessment of study quality using the quality assessment tool for quantitative studies

Author, year	Selection bias	Study design	Confounders	Blinding	Data collection methods	Withdrawals and dropouts	Global rating
Angerer et al., 2015 [36]	weak	moderate	weak	weak	weak	moderate	weak
Braun et al., 2014 [37]	weak	moderate	weak	weak	weak	moderate	weak
Chen et al., 2001 [38]	strong	moderate	weak	weak	moderate	strong	weak
Hankonen et al., 2017 [39]	moderate	strong	strong	moderate	moderate	weak	moderate
Lee et al., 2011 [40]	moderate	strong	strong	moderate	strong	strong	strong
Sickinger et al., 2018 [41]	moderate	moderate	weak	weak	weak	weak	weak
Spook et al., 2016 [42]	moderate	strong	strong	weak	moderate	weak	weak
Verloigne et al., 2017 [43]	moderate	moderate	weak	weak	strong	weak	weak
Walter et al., 2013 [44]	moderate	strong	strong	weak	strong	strong	moderate

### Study characteristics

Table 3 provides an overview of study characteristics in detail. Seven of the nine studies were conducted in Europe: four in Germany and one each in Belgium, Finland, and the Netherlands. Two studies were performed at community colleges in Taiwan. Three German studies took place in workplace settings, while the other European studies were conducted at VET schools. Sample sizes ranged from 23 to 231 participants, with a mean age between 15.5 and 19.4 years.

**Table 3:** Study characteristics

Author, year	Country	Study design	Target group^**a**^; setting	Sample size (n)	Sex	Mean age
Angerer et al., 2015 [36]	Germany	controlled study	overweight apprentices; automobile factory	IG: 60 CG: 32	no data	15–19 (range)
Braun et al., 2014 [37]	Germany	controlled study	young adults with learning impairments; rehabilitation-institution for vocational training	IG: 27 CG: 25	46.2% female	18.9
Chen et al., 2001 [38]	Taiwan	pre-post design	overweight adolescent nursing students; junior college	IG: 55	only female	15.5
Hankonen et al., 2017 [39]	Finland	CRT^b^	vocational students; vocational school unit	IG: 26 CG: 17	85% female	18.9
Lee et al., 2011 [40]	Taiwan	CRT^b^	nursing students; junior college of nursing	IG: 46 CG: 48	only female	16.2
Sickinger et al., 2018 [41]	Germany	pre-post design	trainees in the metal industry; major company in the metal industry	IG: 51^d^	only male	17.0
Spook et al.,2016 [42]	The Netherlands	CRT^c^	secondary vocational education students; vocational education schools	IG: 105 CG: 126	62.8% female	17.2
Verloigne et al.,2017 [43]	Belgium	controlled study	lower-educated girls; vocational and technical schools	IG: 91^e^ CG: 105^e^	only female	16.0
Walter et al.,2013 [44]	Germany	RCT	apprentices; Institute of Technology	IG: 12 CG: 11	52% female	19.4

*CG* control group, *CRT* cluster randomized controlled trial, *IG* intervention group, *RCT* randomized controlled trial

^a^The target group is defined as young adults attending VET. The use of different terminology for VET students (e.g., apprentices or trainees) depends on the respective study. ^b^Four classes of one school/college were randomized. ^c^Four schools were randomized. ^d^*N* = 74 in total, but only men were included in the analysis. ^e^Allocated to three control and intervention schools each

### Intervention characteristics

Intervention details are presented in Table 4, with interventions ranging from 4 weeks to 2 years in duration. Regarding the addressed behavior, the interventions either focused on PA only [37, 39, 40, 43, 44] or followed a multi-behavioral approach in which, for example, alcohol consumption, life-skills training, and/or nutrition were treated in addition to PA [36, 38, 41, 42]. Three interventions comprised multiple components that either addressed a person’s behavior or additionally adjusted the conditions in the setting [36, 39, 43]. For example, Verloigne et al. [43] offered various PA measures, while Angerer et al. [36] and Hankonen et al. [39] modified the context by providing PA equipment. The other six one-component interventions focused solely on individuals’ behavior, comprising stand-alone information and course offerings that included the provision of information or behavioral training (e.g., information, motivation, and counselling).

**Table 4:** Intervention characteristics and study findings

Author, year	Intervention group	Control group	Period	Outcome measure	Effects and effect sizes (d)
Angerer et al., 2015 [36]	“Fit4U”: intensive nutrition counselling, sports facilities, life-skills training, and introduction of health lessons into compulsory education in VET school, provision of sports equipment during breaks; behavior- and environment-oriented measures; multi-behavior (PA, nutrition, life-skills training)	no intervention offered	2 years	**PF:** BMI, cardiopulmonary fitness **PP:** sugar and fat metabolism **PsF:** psychological aspects related to mental health	no significant changes
Braun et al., 2014 [37]	one hour of individually adapted circuit training with endurance and strength training components once a week; single behavior (PA only)	compulsory physical education	1 year	**PF:** aerobic step test, coordination, flexibility, BMI; **PP:** blood pressure, heart rate; **PsF:** self-rating of physical and mental health characteristics	**PF:** significant increase in the number of steps (*d*_corr_ = 1.17) and duration of the step test (*d*_corr_ = 0.74)
Chen et al., 2001 [38]	Health Promotion Counselling: total of 8 h of whole group education (nutrition behavioral change, exercise behavior modifications, instruction on physiological side effects of being overweight and the benefits of weight reduction, life appreciation, interpersonal support and stress management – 2 h each), additional 12 h of small-group health promotion counselling; multi-behavior (PA, nutrition)	no control group	1 year	**PA:** exercise behavior **PF:** body weight, WLI **PP:** blood pressure, HDL, LDL, TG, TC	**PA:** significant increase in PA level (*d* = 0.74) **PF:** significant decrease in body weight (*d* = 0.21) and WLI (*d* = 0.28) **PP:** significant decrease in systolic pressure (*d* = 0.87), HDL (*d* = 0.77) and TC (*d* = 0.26)
Hankonen et al., 2017 [39]	“Let’s Move It”: 6 h of group-based intervention for students, two 2-h training workshops for teachers to reduce their students’ sitting in class, physical choice architecture (providing PA equipment to enable light PA in classrooms); individual and environmental changes; participatory approach involving stakeholders in stepwise intervention development; single behavior (PA only)	standard care, i.e., normal curriculum plus a leaflet on recommendations for youth PA	5 weeks	**PA:** moderate-to-vigorous PA **PF:** body composition **PsF:** self-reported use of behavior change technique	**PsF:** significant increase in use of behavior change technique (0.74 < *d*_corr_ < 0.90)
Lee et al., 2011 [40]	“SPAA-G”: original content and activity in a physical education class, plus school-based PA intervention for adolescent girls program, combining the theoretical foundation of self-efficacy theory and provision of a pedometer; single behavior (PA only)	original content and activity in a physical education class	12 weeks	**PA:** aerobic step test **PF:** cardiopulmonary endurance **PsF:** perceived self-efficacy	**PA:** significant increase in the number of steps (*d*_corr_ = 0.78)
Sickinger et al., 2018 [41]	12 theoretical and practical teaching units between 65 and 90 min each on the topics of nutrition, alcohol and nicotine consumption, and PA during VET; participatory approach involving 6 focus groups in the sensitization unit; multi-behavior (PA, nutrition, alcohol and nicotine consumption)	no control group	11 months	**PA:** at least 1 h of PA per day **PsF:** general self-effectiveness expectations	**PsF:** significant increase in general self-effectiveness expectations (*d* = 0.27)
Spook et al.,2016 [42]	“Balance It”: serious self-regulation game designed to target dietary intake and PA; this tailored, interactive multimedia game could be played at any time and place desired on a daily basis, entailing a combination of behavior change techniques derived from self-regulation theory with serious game elements; multi-behavior (PA, nutrition)	waiting list control group: no intervention between measures	4 weeks	**PA:** moderate PA, vigorous PA, active transport **PsF:** determinants of PA	no significant changes
Verloigne et al.,2017 [43]	specific interventions were developed by a co-creation group; several co-creation sessions during lunch break (about 50 min); group brainstormed on what it could do to change specific behaviors and ascertain what might be relevant for the girls in their school; co-creational approach; single behavior (PA only)	control schools did not receive any information on PA or health outside the normal curriculum	6 months	**PA:** time spent in PA **PsF:** self-efficacy, perceived benefits of PA, perceived barriers to be physically active	**PA:** significant increase in extracurricular sports participation (*d* = 0.19) **PsF:** significant intervention effect on self-efficacy (*d* = 0.63)
Walter et al.,2013 [44]	aerobic endurance intervention: instructed outdoor running training twice a week; initial duration of 30 min was increased continuously to 60 min over 10 weeks; single behavior (PA only)	instructed not to alter their PA and exercise patterns during the control period	10 weeks	**PA:** mean activity intensity **PF:** aerobic endurance capacity **PsF:** mood state	**PA:** significant change in mean activity intensity (*d* = 0.87) **PF:** significant change in aerobic endurance capacity (1.03 < *d* < 1.40)

*BMI* body mass index, HDL high-density lipoprotein, *LDL* low-density lipoprotein, *PA* physical activity, *PF* physical fitness, *PP* physiological parameters, *PsF* psychological factors, *TC* total serum cholesterol, *TG* triglycerides, *VET* vocational education and training, *WLI* weight-for-length index

Furthermore, the interventions differed in the way they were developed and implemented. Essentially, the interventions could be classified into top-down and bottom-up interventions. Top-down interventions were developed and implemented by experts and followed a theoretical and scientific orientation in terms of their goals and content [36–38, 40, 42, 44]. By contrast, the bottom-up interventions followed a participatory approach, ranging from the target group’s involvement in designing teaching units [41], through a stepwise intervention development involving different stakeholders [39], to the entire intervention development and implementation using a co-creation approach [43].

Further special characteristics of individual studies included, for example, an online-based intervention in the form of a multimedia game [42] or an additional intervention for teachers to reduce their students’ sedentary behavior in class [39].

### Study findings

The studies’ outcomes are grouped into four major categories: PA, physical fitness, physiological parameters, and psychological factors. Most studies measured more than one of these outcome categories.

Seven studies measured PA either subjectively using standardized questionnaires or objectively using accelerometers. Four of the seven studies [38, 40, 43, 44] found significant baseline to post-intervention improvements in PA. Among these, two studies subjectively measured PA and identified a significant intervention effect on activity level [38] and extracurricular sports participation [43], while two studies objectively measured PA and found significant effects. Thus, Lee et al. [40] revealed a significant increase in the number of aerobic steps, and Walter et al. [44] indicated a significant increase in mean activity intensity. Three studies did not find significant changes in PA level [39, 41, 42].

Physical fitness components were tested by motor performance tests or body analyses in six studies. Two of these studies identified a significant intervention effect on endurance [37, 44]. In another study, a significant decrease in body weight and weight-for-length index was found following the intervention [38]. The remaining three studies found no significant changes in body mass index, body composition, or cardiopulmonary endurance [36, 39, 40].

Physiological parameters measured through blood pressure or blood tests were examined in three studies. Only Chen et al. [38] reported significant improvements from baseline to post-intervention on physiological parameters, in this case systolic blood pressure, high-density lipoprotein, and total serum cholesterol. In two other studies, no significant effects on blood pressure, heart rate, sugar metabolism, or fat metabolism were found [36, 37].

Eight studies assessed psychological factors using standardized questionnaires. Of these, three identified a significant change in psychological factors. Hankonen et al. [39] reported a significant improvement in the use of behavior change techniques from baseline to post-intervention in the intervention group. Furthermore, Sickinger et al. [41] found significant improvements in general self-effectiveness expectations, and Verloigne et al. [43] reported a significant intervention effect on self-efficacy. Five studies did not find significant changes in psychological factors, including determinants of PA, mood state, psychological aspects related to mental health, self-efficacy, or self-rating of physical and mental health characteristics [36, 37, 40, 42, 44].

Overall, two studies indicated significant effects in all measured outcome variables [38, 43], whereas two other studies did not find significant effects in any measured outcome variables [36, 42].

## Discussion

To our knowledge, this is the first systematic review to identify PA-promoting interventions in VET and to examine their effects on PA-related outcomes such as PA level, physical fitness, physiological parameters, or psychological factors. In total, nine studies met the inclusion criteria, covering a broad range of interventions and outcomes measured. All but two studies found significant improvements for at least one PA-related outcome, with the majority of studies indicating a mix of both significant and non-significant effects. These heterogeneous effects, coupled with the overall weak study quality, limited our ability to draw clear conclusions about the potentially most effective intervention strategies.

An existing problem, confirmed in our review, is the lack of studies dealing with the promotion of PA in VET. As already assumed, very few studies have focused on this issue, and unfortunately, those that are available are of poor quality. Ensuring high study quality while simultaneously taking local requirements and conditions into account is particularly difficult in real-world settings [46]. Accordingly, methodologically complex and comprehensive studies are necessary to better examine interventions’ effectiveness in the VET field [38]. Regarding the countries in which the studies were carried out, it is striking that all but two Taiwanese studies were conducted in Europe, whereas Anglo-American studies were completely absent. One reason for this could be the large number of occupations with a required VET qualification and the associated high importance of a formal curriculum-based VET in European countries, such as the Benelux and Scandinavian countries or, in particular, Germany [13]. However, pursuing higher education by enrolling in colleges or universities is the most common pathway after graduating from high school in the U.S. [47]. Thus, the lack of studies is astonishing and also indicates a research gap in physical activity promotion with VET students. In summary, the evidence for PA promotion in the VET context is sparse, comprising data mainly only from European studies. Furthermore, due to VET systems’ heterogeneity in Europe, our findings cannot be generalized.

Nevertheless, we tried to identify further conspicuous aspects and similarities regarding intervention characteristics, such as their approaches, components, and content. One remarkable result that emerged from our review is that both bottom-up and top-down interventions revealed positive effects. Taking a closer look, interventions designed with a participatory bottom-up approach tended to improve relevant psychological factors related to PA, such as self-efficacy or the use of behavior change techniques, but not PA levels per se [39, 41, 43]. In contrast, none of the five top-down interventions that measured psychological factors indicated positive effects for this set of outcomes [36, 37, 40, 42, 44]. Out of a total of six studies using top-down interventions, four reported significant improvements in PA level, physical fitness, and/or physiological parameters [37, 38, 40, 44]. Thus, top-down interventions seem to improve outcomes on the physical level. According to existing literature, “traditional” interventions designed using a top-down approach have shown limited success and have been criticized for their lack of long-term sustainability [48–50]. A possible reason for these interventions’ failure may be a lack of consideration of the complex influence of different factors between the individual and environment.

To counteract this problem, bottom-up interventions seem to be promising. With a participatory or co-creational approach, it is possible to develop interventions tailored to the needs of the target group and given setting, thereby increasing acceptance by the target group and facilitating the intervention’s sustainability [49, 51, 52]. From our perspective, we cannot estimate the long-term effectiveness and sustainable implementation of these interventions, as no long-term follow-ups or reports on the continuation and anchoring of the interventions exist. Therefore, it is not yet possible to conclude which approach is more appropriate; the tendency for bottom-up approaches to improve outcomes on the psychological level and for top-down approaches to improve outcomes on the physical level needs to be investigated in further studies. In such studies, recommendations for the intervention research process made by van Sluijs et al. [52, 53] also should be taken into account. The authors report an apparent willingness among adolescents to increase PA as evidenced by an expressed desire to do more types of PA more often. In reality, this intention often fails due to difficulty translating intention to action. Therefore, it is necessary to transform this enthusiasm into effective PA-promoting interventions [53]. Active engagement in the form of participatory or co-creational approaches could be a key to success in developing acceptable and attractive interventions [52].

Recent literature recommends multi-component interventions that combine various measures to promote PA, e.g., behavioral, educational, and/or environmental elements [22, 51, 54]. In particular, combining individual and environmental changes is also acknowledged in other studies, as effective behavioral changes in individuals require supportive policies and environments [55, 56]. Although our data are not strictly conclusive, the two studies showing significant effects in PA level and psychological factors [39, 43] seem to support this approach. With several comprehensive measures, it is possible to extend the target group’s reach and create a PA-friendly environment. To achieve this, PA intervention components of the examined studies included offering various PA programs, providing PA equipment, and conducting workshops for teachers as experts for PA promotion. In our case, the two studies in which multi-component interventions yielded significant effects also were interventions developed based on a participatory or co-creational approach. Therefore, the use of a bottom-up approach may be a promising strategy to create diverse and comprehensive PA-promoting measures that consider both the individual and environment.

Prior studies have discussed the benefits and effectiveness of multi-behavior interventions compared with single-behavior interventions. In theory, it is assumed that different unhealthy behaviors co-occur and are mutually dependent. Therefore, targeting more than one behavior, rather than just a single behavior, could lead to greater health benefits through lifestyle changes [57, 58]. However, in practice, such multi-behavioral interventions have proved to be an obstacle to success [22, 59, 60]. Our review demonstrated mixed results in terms of single-behavioral and multi-behavioral interventions’ effectiveness. Two studies reported positive results from their multi-behavioral interventions [38, 41], while the only two studies that showed no significant effects also comprised multi-behavioral interventions [36, 42]. In contrast, all studies that focused only on PA behavior revealed significant effects on at least one outcome. On the basis of our review, multi-behavioral interventions can work, but changing multiple health behaviors simultaneously also can lead to excessive demands and burdens on participants and thus reduce the interventions’ effectiveness [57].

Against this backdrop and the results of our review, the VET field seems to be promising for the implementation of PA-promoting interventions, as many young people can be reached in an environment where a time structure and organizational framework are provided. Our review highlights the positive, yet inconsistent, effects from PA-promoting interventions in VET. Thus, it is difficult for us to declare explicit practical recommendations based only on our results. Moreover, the different VET systems and, consequently, the various contextual factors make it difficult to interpret our results. In future studies, clearer reporting on the intervention and, in particular, on the contextual factors would be helpful to provide substantive implications and recommendations [61]. Nevertheless, regarding the results from van Sluijs et al. [52, 53], multi-component interventions tailored to the target group and context seem to be a good way to increase acceptance and participation and, thus, interventions’ effectiveness. To understand interventions’ effectiveness and make further recommendations regarding the development and implementation of PA interventions in VET, high-quality studies with long-term follow-ups conducted in real-world settings are needed.

### Limitations

Our review’s limitations are influenced by the number and quality of the included studies. First, we limited our search to studies published in English and German between 2000 and 2018 and did not search for grey literature. We may have been able to find some studies without these restrictions, but we believe that we found the core studies through our extensive search. Second, we identified some weaknesses in the assessment of study quality using the EPHPP tool. The rigid scoring system may not always distinguish more robust studies from weaker ones. In particular, the lack of blinding was often the crucial factor for the studies’ weak global ratings. This is in line with other studies that have reported on the challenges of blinding in behavioral interventions [62, 63]. Finally, due to the small number of included studies, overall weak study quality, and heterogeneity of outcome measures, we were unable to conduct a meta-analysis. Thus, it should be taken into account that the conclusions on the linkage between intervention characteristics and intervention effectiveness presented in this systematic review are based on a descriptive, rather than a quantitative, analysis.

## Conclusion

The present systematic review provides detailed insight into literature concerning the effectiveness of interventions that promote PA in VET. First, with most of the examined studies revealing significant improvements in at least one PA-related outcome, PA interventions have the potential to be efficacious in VET. However, the results are inconclusive, as most studies indicated a mix of both significant and non-significant effects. Second, in contrast to the numerous studies on PA interventions in young people, only a few published studies feature PA interventions that are targeted specifically at VET students. Third, it has become clear that the available studies are mainly from the European and Asian regions. A global perspective on the topic is therefore not yet possible at present. Thus, these results also underline the need for further research in this new research area. In addition to addressing this current lack of studies, future research should focus on high-quality studies with long-term follow-ups. Only in this way is it possible to take a closer look at PA promotion in VET, to draw clear conclusions about the effectiveness of studies, to make recommendations for practical use, and, thus, to answer more precisely the question of what we know about physical activity interventions in VET.

## Supplementary information

**Additional file 1:** Completed PRISMA checklist.

**Additional file 2:** Search strategy.

## Data Availability

All data used to derive the study findings are included in this published article and its additional files.

## Abbreviations

BMI: Body mass index
CG: Control group
CRT: Cluster randomized controlled trial
EPHPP: Effective Public Health Practice Project
HDL: High-density lipoprotein
IG: Intervention group
LDL: Low-density lipoprotein
PA: Physical activity
PF: Physical fitness
PP: Physiological parameters
PsF: Psychological factors
RCT: Randomized controlled trial
SES: Socioeconomic status
TC: Total serum cholesterol
TG: Triglycerides
VET: Vocational education and training
WLI: Weight-for-length index
