# Supplementary material for: What do we know about physical activity interventions in vocational education and training? A systematic review
Source: BMC Public Health. 2020 Jun 22;20:978. doi: 10.1186/s12889-020-09093-7 (PMC7309979; doi:10.1186/s12889-020-09093-7)
Supplement: Supplementary file 2 — Additional file 2: Search strategy. [file 12889_2020_9093_MOESM2_ESM.pdf]

Additional File 2: Search strategy

List of search terms

Setting / population	Health behavior of interest	Type of study
apprentice*<br>trainee<br>trainees<br>traineeship<br>((student OR students) NOT (university OR “primary school” OR “elementary school” OR “high school” OR child OR children))<br>“vocational education”<br>“vocational training”<br>“vocational school”<br>“job training”<br>“professional training”<br>“professional education”<br>"postsecondary school"<br>"post secondary school"<br>"post-secondary school"<br>"occupational education"	“physical activity”<br>exercise<br>exercises<br>exercising<br>sport<br>sports<br>fitness	intervention<br>measure<br>measures<br>program<br>programs<br>programme<br>programmes<br>treatment<br>evaluation<br>evaluating<br>evaluate

Electronic search strategy used in PubMed

Date: August 22, 2018
Database: PubMed
Filters: Journal Article; Publication date from 2000/01/01 to 2018/12/31; English; German

#	Search string	Results
1	apprentice* [tiab]	1,177
2	(apprentice* [tiab] OR trainee* [tiab])	17,516
3	(apprentice* [tiab] OR trainee [tiab] OR trainees [tiab] OR traineeship [tiab])	17,405
4	(apprentice* [tiab] OR trainee [tiab] OR trainees [tiab] OR traineeship [tiab] OR student [tiab] OR students [tiab])	162,371
5	(student [tiab] OR students [tiab])	147,723
6	((student [tiab] OR students [tiab]) NOT (university [tiab] OR “primary school” [tiab] OR “elementary school” [tiab] OR “high school” [tiab] OR child [tiab] OR children [tiab]))	97,572
7	"vocational education" [mh]	381

8	(apprentice* [tiab] OR trainee [tiab] OR trainees [tiab] OR traineeship [tiab] OR ((student [tiab] OR students [tiab]) NOT (university [tiab] OR “primary school” [tiab] OR “elementary school” [tiab] OR “high school” [tiab] OR child [tiab] OR children [tiab])) OR “vocational education” [tiab] OR “vocational training” [tiab] OR “vocational school” [tiab] OR “job training” [tiab] OR “professional training” [tiab] OR “professional education” [tiab] OR "vocational education" [mh])	116,694
9	"physical activity"	80,783
10	"physical activity" [tiab]	75,924
11	("physical activity" [tiab] OR exercise [tiab])	204,606
12	exercise [mh]	329,810
13	(apprentice* [tiab] OR trainee [tiab] OR trainees [tiab] OR traineeship [tiab] OR ((student [tiab] OR students [tiab]) NOT (university [tiab] OR “primary school” [tiab] OR “elementary school” [tiab] OR “high school” [tiab] OR child [tiab] OR children [tiab])) OR “vocational education” [tiab] OR “vocational training” [tiab] OR “vocational school” [tiab] OR “job training” [tiab] OR “professional training” [tiab] OR “professional education” [tiab] OR "vocational education" [mh]) AND (“physical activity” [tiab] OR exercise [tiab] OR exercises [tiab] OR exercising [tiab] OR sport [tiab] OR sports [tiab] OR fitness [tiab] OR exercise [mh])	7,220
14	(intervention [tiab] OR measure [tiab] OR measures [tiab] OR program [tiab] OR programs [tiab] OR programme [tiab] OR programmes [tiab] OR treatment [tiab] OR evaluation [tiab] OR evaluating [tiab] OR evaluate [tiab])	4,360,967
15	((apprentice* [tiab] OR trainee [tiab] OR trainees [tiab] OR traineeship [tiab] OR ((student [tiab] OR students [tiab]) NOT (university [tiab] OR “primary school” [tiab] OR “elementary school” [tiab] OR “high school” [tiab] OR child [tiab] OR children [tiab])) OR “vocational education” [tiab] OR “vocational training” [tiab] OR “vocational school” [tiab] OR “job training” [tiab] OR “professional training” [tiab] OR “professional education” [tiab] OR "vocational education" [mh]) AND (“physical activity” [tiab] OR exercise [tiab] OR exercises [tiab] OR exercising [tiab] OR sport [tiab] OR sports [tiab] OR fitness [tiab] OR exercise [mh]) AND (intervention [tiab] OR measure [tiab] OR measures [tiab] OR program [tiab] OR programs [tiab] OR programme [tiab] OR programmes [tiab] OR treatment [tiab] OR evaluation [tiab] OR evaluating [tiab] OR evaluate [tiab]))	3,911
16	(("postsecondary school" [tiab] OR "post secondary school" [tiab] OR "post-secondary school" [tiab] OR "occupational education" [tiab]) AND (physical activity” [tiab] OR exercise [tiab] OR exercises [tiab] OR exercising [tiab] OR sport [tiab] OR sports [tiab] OR fitness [tiab] OR exercise [mh]) AND (intervention [tiab] OR measure [tiab] OR measures [tiab] OR program [tiab] OR programs [tiab] OR programme [tiab] OR programmes [tiab] OR treatment [tiab] OR evaluation [tiab] OR evaluating [tiab] OR evaluate [tiab]))	0
